# Single-molecule techniques in studying the molecular mechanisms of DNA synapsis in non-homologous end-joining repair

**DOI:** 10.52601/bpr.2024.240043

**Published:** 2025-02-28

**Authors:** Yuhao Jiang, Chao Zhao, Chenyang Zhang, Weilin Li, Di Liu, Bailin Zhao

**Affiliations:** 1 Department of Hematology, The Second Affiliated Hospital of Xi’an Jiaotong University, Xi'an 710004, China; 2 Institute of Molecular and Translational Medicine (IMTM), and Department of Biochemistry and Molecular Biology, Xi'an Jiaotong University Health Science Center, Xi'an 710061, China; 3 Institute of Biomedical and Health Engineering, Shenzhen Institute of Advanced Technology, Chinese Academy of Sciences, Shenzhen 518055, Guangdong, China

**Keywords:** Single-molecule techniques, smFRET, Magnetic tweezers, Non-homologous end joining (NHEJ), DNA Synapsis

## Abstract

DNA double-strand breaks (DSBs) are the most severe form of DNA damage, primarily repaired by the non-homologous end joining (NHEJ) pathway. A critical step in this process is DNA synapsis, where the two broken ends are brought together to facilitate timely repair. Deficiencies in NHEJ synapsis can lead to improper DNA end configurations, potentially resulting in chromosomal translocations. NHEJ synapsis is a highly dynamic, multi-protein mediated assembly process. Recent advances in single-molecule techniques have led to significant progress in understanding the molecular mechanisms driving NHEJ synapsis. In this review, we summarize single-molecule methods developed for studying NHEJ synapsis, with a particular focus on the single-molecule fluorescence resonance energy transfer (smFRET) technique. We discuss the various molecular mechanisms of NHEJ synapsis uncovered through these studies and explore the coupling between synapsis and other steps in NHEJ. Additionally, we highlight the strategies, limitations, and future directions for single-molecule studies of NHEJ synapsis.

## INTRODUCTION

Cellular DNA is vulnerable to various types of damage during cell growth and development, with double-strand breaks (DSBs) being the most severe form (Chen *et al.* 2021). DSBs occur when both complementary strands of the DNA molecule break at nearby positions, disrupting the continuity and integrity of the genome. This disruption can impair the transmission and expression of genetic information, potentially driving tumorigenesis (Liu and Lieber 2022; Liu *et al.* 2021) and cell death (Bunting and Nussenzweig 2013). DSBs inside the cells are mainly repaired through four pathways: non-homologous end joining (NHEJ), homologous recombination (HR), single-strand annealing (SSA), and alternative end-joining (a-EJ) (Chang *et al.* 2017; Lieber 2023; Zhao *et al.* 2020a). NHEJ is the predominant repair mechanism for DSBs in mammalian cells (Karanam *et al.* 2012) and plays crucial roles in the adaptive immune system (Chang *et al.* 2017; Wang *et al.* 2020; Zhao *et al.* 2020a) and CRISPR-Cas9-mediated genome editing (Xue and Greene 2021; Zhang *et al.* 2024). The NHEJ process consists of three primary steps: end synapsis, processing, and ligation (Zhao *et al.* 2020a). The three steps are not independent but interconnected to a certain degree (Chang *et al.* 2017; De Bragança *et al.* 2023b; Watanabe and Lieber 2023; Zhao *et al.* 2020a).

The process of bringing the broken ends into close proximity prior to the ligation of either strand, referred to as synapsis, is critical for NHEJ repair. NHEJ synapsis is a complex and highly dynamic process involving multiple proteins (Graham *et al.* 2016; Graham *et al.* 2018; Wang *et al.* 2018; Zhao *et al.* 2020a; Zhao *et al.* 2019). Ensemble pull-down assays (Andres *et al.* 2012; DeFazio *et al.* 2002; Ramsden and Gellert 1998) and electronic microscopy (EM) analyses (Andres *et al.* 2012; DeFazio *et al.* 2002; Grob *et al.* 2012) have attempted to identify the essential components for NHEJ synapsis. However, the pull-down assay cannot capture synaptic intermediates, and both pull-down and EM methods lack dynamic information on the sequential assembly of synaptic complexes. The highly dynamic nature of NHEJ synapsis makes single-molecule methods particularly well-suited for its study. Recent advances in single-molecule techniques have enabled significant progress in characterizing the dynamic nature of NHEJ synapsis and the intermediates formed during this process (Graham *et al.* 2016; Graham *et al.* 2018; Wang *et al.* 2018; Zhao *et al.* 2019). These techniques have also shed light on the coupling mechanisms between synapsis and the other two steps of NHEJ (De Bragança *et al.* 2023b; Kong and Greene 2021; Stinson *et al.* 2020; Zhao *et al.* 2020b; Zhao *et al.* 2019). In this review, we provide an overview of the biochemical processes involved in NHEJ repair. We focus particularly on single-molecule techniques, especially the single-molecule fluorescence resonance energy transfer (smFRET), which have been successfully applied to study NHEJ synapsis. We also discuss recent insights into the dynamic nature of NHEJ synapsis and its coupling with other repair steps, as revealed through single-molecule studies.

## OVERVIEW OF THE BIOCHEMICAL PROCESSES OF NHEJ REPAIR

**Figure 1:**
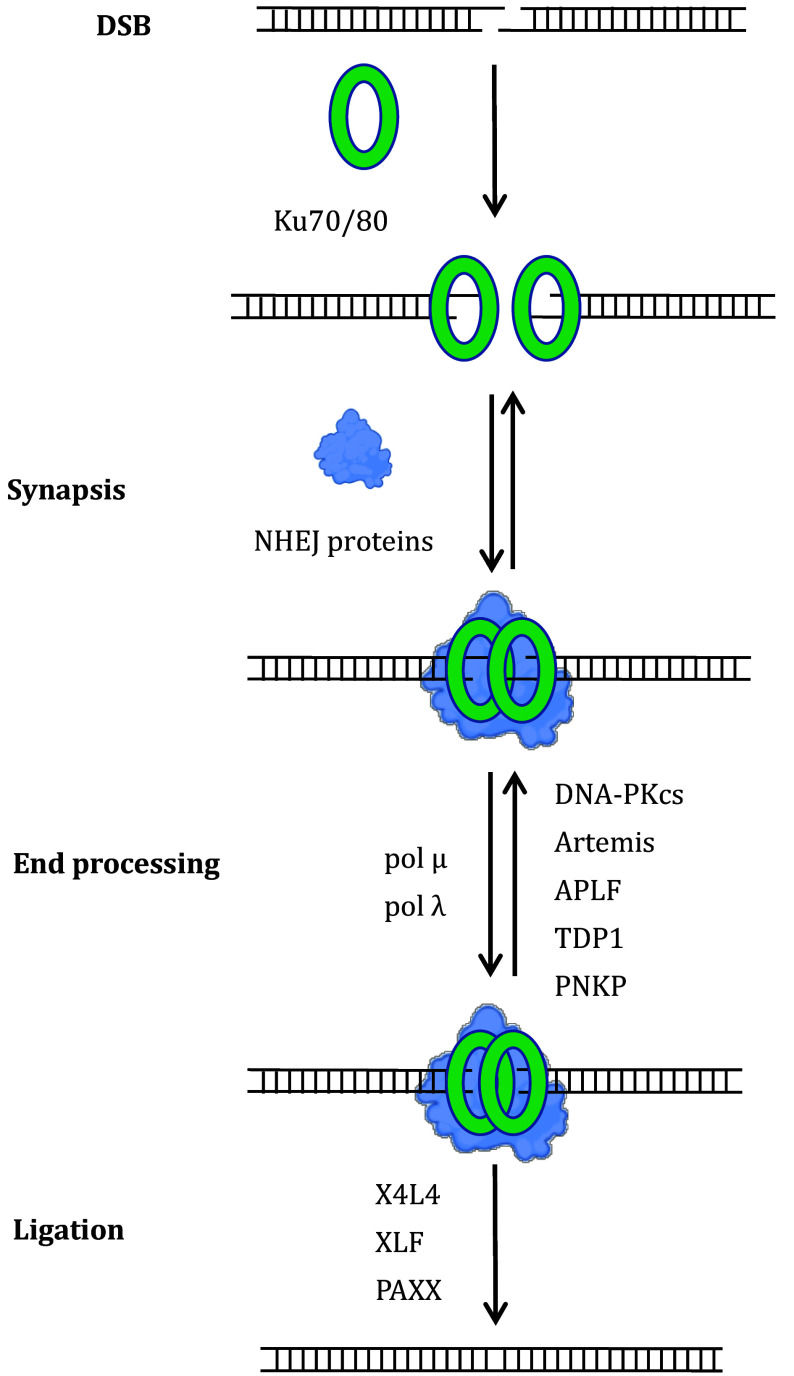
Three biochemical steps of the non-homologous DNA end-joining (NHEJ) process. This figure outlines the key stages of the NHEJ repair mechanism following DNA double-strand breaks. The process can be divided into three main steps: end synapsis, processing, and ligation. First, the Ku heterodimer recognizes the broken DNA ends, initiating the recruitment of NHEJ proteins that bridge and stabilize the damaged DNA, leading to the formation of a synaptic complex. In most cases, the DNA ends require processing by nucleases (Artemis and APLF), polymerases (pol μ and pol λ), or other modifiers (TDP1 and PNKP) to reveal microhomology between the ends. Once the DNA ends are properly aligned and compatible for ligation, the X4L4 complex completes the repair by sealing the ends

Biochemically, NHEJ repair consists of three main steps: end synapsis, processing, and ligation (Fig. 1). Following DSB formation, the Ku70-Ku80 (Ku70/80) heterodimer recognizes and binds to the double-stranded DNA ends, forming a ring-like structure with a high affinity for the broken ends (Walker *et al.* 2001). Ku70/80 then directly or indirectly recruits other NHEJ proteins that function in different stages of the repair process. The first essential step, end synapsis, ensures the correct alignment of DNA ends to prevent chromosomal translocations caused by the random diffusion of broken ends.

In most cases, the DSB ends cannot be ligated immediately and require processing to create ligation-compatible ends. End processing relies on the collaborative actions of the kinase—DNA-dependent protein kinase catalytic subunit (DNA-PKcs), nuclease—Artemis, and aprataxin and PNKP like factor (APLF), DNA polymerase—polymerase μ (Pol μ) and polymerase λ (Pol λ), and the nucleotide modifier—tyrosyl-DNA phosphodiesterase 1 (TDP1) and polynucleotide kinase 3’-phosphatase (PNKP). These proteins modify the DNA ends, enabling the next step, ligation. The XRCC4-Ligase4 complex (X4L4) is essential for ligation in NHEJ and is aided by XRCC4-like factor (XLF) and paralogue of XRCC4 and XLF (PAXX) (Chang *et al.* 2017; Graham *et al.* 2018; Wang *et al.* 2018; Zhao *et al.* 2019, 2020a).

The three stages of NHEJ are not entirely independent but dynamically coupled. The events occurring in one step can influence the events of the following stages. Single-molecule techniques have allowed us to explore this dynamic coordination in more detail, shedding light on the intermediates and transition states formed during synapsis and their coupling with the processing and ligation steps (De Bragança *et al.* 2023b; Graham *et al.* 2016; Kong and Greene 2021; Stinson *et al.* 2020; Wang *et al.* 2018; Zhao *et al.* 2019, 2020b).

## SINGLE-MOLECULE TECHNIQUES APPLIED FOR STUDYING NHEJ SYNAPSIS

Among the different biochemical stages of NHEJ repair, end synapsis is the most challenging to study due to its reversible and dynamic nature. Traditional biochemical methods, such as PAGE assays, are inadequate for capturing these transient intermediates. Single-molecule techniques have revolutionized this field by enabling real-time observation of NHEJ synapsis dynamics. Combined with the insights of ensemble methods, single-molecule methods have facilitated the understanding of the underlying mechanisms of the synapsis process by identifying multiple synaptic intermediates (De Bragança *et al.* 2023b; Graham *et al.* 2016; Kong and Greene 2021; Stinson *et al.* 2020; Wang *et al.* 2018; Zhao *et al.* 2019, 2020b).

Single-molecule techniques used for studying NHEJ synapsis can be broadly classified into fluorescence-based (Graham *et al.* 2016; Reid *et al.* 2015; Stinson *et al.* 2020; Zhao *et al.* 2019, 2020b) and mechanical methods (De Bragança *et al.* 2023a; Wang *et al.* 2018). Fluorescence methods, including single-molecule fluorescence resonance energy transfer (smFRET) and fluorescence colocalization assays, are particularly useful for studying protein-DNA interactions and dynamic processes such as synapsis. Mechanical methods, including magnetic tweezers (De Bragança *et al.* 2023a; Wang *et al.* 2018) and optical tweezers (Brouwer *et al.* 2016) have also been used in the study of the NHEJ repair process.

### Fluorescence methods for studying NHEJ synapsis

Single-molecule fluorescence techniques, particularly smFRET, are frequently applied to study the interactions between biological macromolecules, such as protein folding, enzyme kinetics, and nucleic acid-protein interactions (Feng *et al.* 2021; Ha *et al.* 1996; Roy *et al.* 2008). The capability of monitoring interactions at the single-molecule level makes it ideal for examining the dynamics of DNA ends mediated by NHEJ repair proteins.

**Figure 2:**
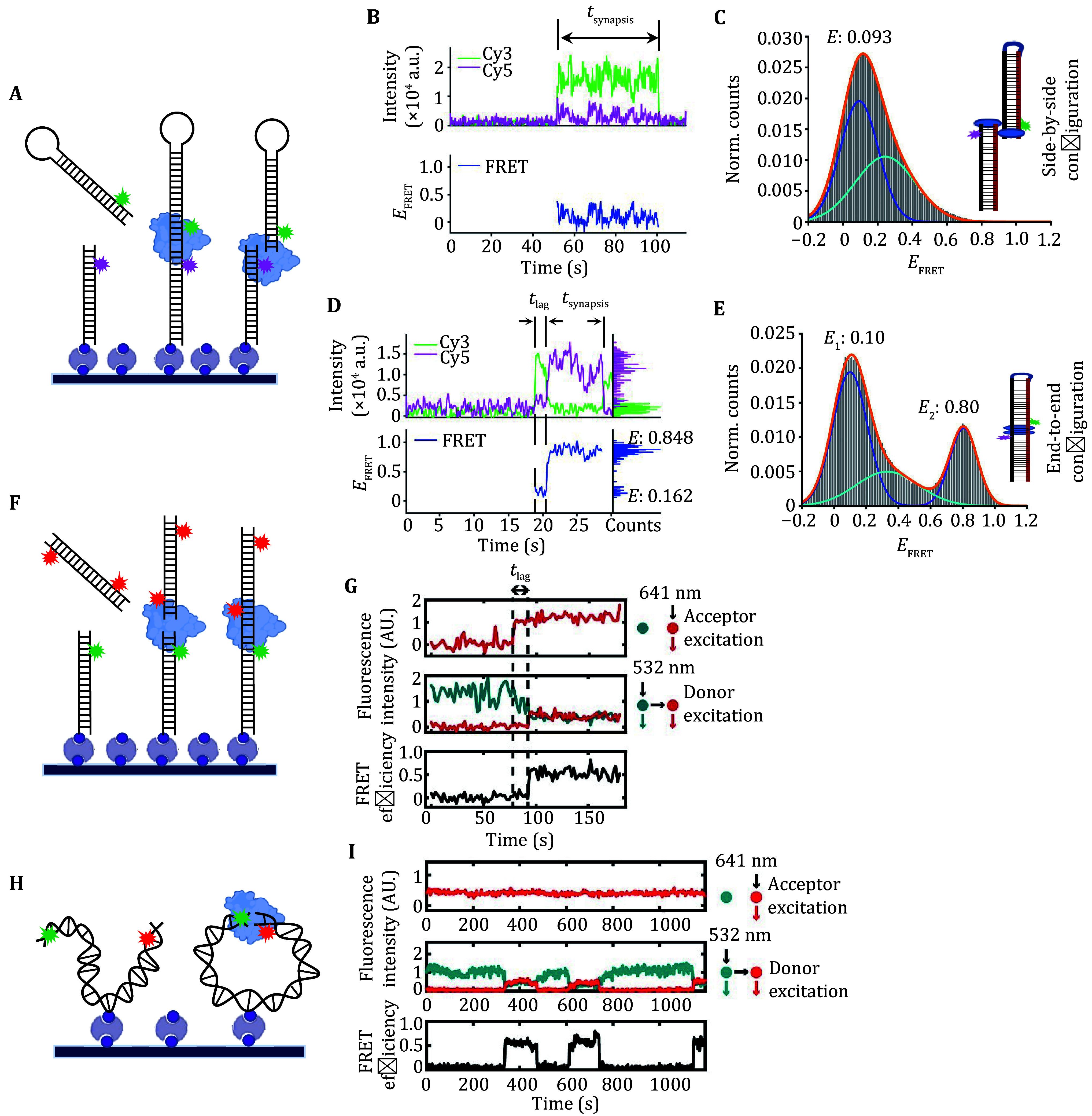
Single-molecule fluorescence strategies applied for studying NHEJ synapsis. This figure illustrates how single-molecule Förster resonance energy transfer (smFRET) assays are utilized to investigate the synapsis process in non-homologous end joining (NHEJ). **A** A slide-immobilized DNA strand is labeled with a Cy5 dye (acceptor) positioned four base pairs from the end. The solution DNA is labeled at one end with a Cy3 dye (donor), while the opposite end is covalently linked to a small loop, preventing synapsis at that end. **B**–**E** Trajectories of donor intensity, acceptor intensity, and FRET level (**B** and **D**), and the FRET distribution of formed synaptic complexes (**C** and **E**) in studies using looped solution DNA. The appearance of a FRET signal marks the occurrence of synapsis. A low, fluctuating FRET signal indicates the formation of a flexible synaptic (FS) complex (**B** and **C**), while a high FRET signal close to 0.8 indicates the formation of a close synaptic (CS) complex (**D** and **E**). A transition from low to high FRET levels signifies the conversion of synaptic complexes from the flexible synaptic (FS) state to the close synaptic (CS) state (**D**). **F**,**G** The same labeling strategy as in Panel A is used for the immobilized DNA, with the solution DNA tagged with Cy5 dyes near both ends. This setup allows monitoring of synapsis between the immobilized DNA and either end of the solution DNA. Excitation with alternating 532-nm and 641-nm lasers is applied (**G**). The appearance of an acceptor signal indicates synapsis, and FRET signal fluctuations suggest structural changes within the synaptic complex. **H**,**I** An intramolecular DNA construct is used in the smFRET assay to study synapsis. The donor and acceptor dyes are positioned at opposite ends, with a biotin tag in the middle to immobilize the DNA (**H**). Similar to Panel F, alternating laser excitation is applied. A high FRET signal indicates the formation of the close synaptic complex (**I**). Adapted from Zhao *et al*. 2019 and Graham *et al*. 2016

To study NHEJ synapsis, two DNA duplexes or ends must be fluorescently labeled with donor and acceptor dyes, respectively (Fig. 2). The FRET signal is monitored through the total internal reflection fluorescence (TIRF) microscopy, where the FRET level reflects the distance between the donor and acceptor dyes, thereby indicating the distance between the two DNA ends (Zhang *et al.* 2021). Fluctuations in FRET imply changes in the distance between the duplexes, allowing real-time monitoring of synapsis (Reid *et al.* 2015, 2017; Zhao *et al.* 2019). High FRET values indicate close proximity between the DNA ends, while low values suggest separation. The fluctuations of smFRET reveal dynamic interactions between the two ends. As such, smFRET assays can identify synaptic intermediates, track transitions between different states, and provide valuable kinetic and thermodynamic insights into the role of NHEJ proteins in synapsis (Zhao *et al.* 2019).

For effective smFRET studies of NHEJ synapsis, DNA probes must be designed to capture interactions near the DNA ends. Currently, two main types of DNA probes are used in these studies, which we classified as intermolecular and intramolecular types here (Graham *et al.* 2016; Reid *et al.* 2015; Stinson *et al.* 2020; Zhao *et al.* 2019, 2020b) (Fig. 2). The intermolecular type involves two short DNA oligos — one immobilized on an imaging slide and the other in solution — to monitor intermolecular synapsis (Graham *et al.* 2016; Reid *et al.* 2015; Zhao *et al.* 2019) (Figs. 2A and 2F). These oligos are typically 75–100 bp in length (Graham *et al.* 2016; Reid *et al.* 2015; Zhao *et al.* 2019), similar to the free DNA length between nucleosomes during chromosomal breaks in cells (Aparicio *et al.* 2014). After the first DNA is immobilized on the slide, the solution DNA is introduced along with NHEJ proteins to initiate synapsis. Since NHEJ proteins function primarily at the DNA ends, the donor and acceptor fluorescent labels are placed close to these ends, allowing the FRET signal to reflect interactions at the broken ends. However, since NHEJ proteins can bind to either free end of the probe, synapsis between any unlabeled ends reduces the observed synapsis efficiency. The immobilized probe typically has one free end (Figs. 2A and 2F), while the solution probe has two. To improve the observed efficiency, one strategy involves covalently linking a small loop to one end of the solution probe, leaving the other free for synapsis (Reid *et al.* 2015; Zhao *et al.* 2019) (Fig. 2A). Another approach is to tag both ends of the solution probe with acceptors, allowing synapsis to be monitored at either end (Graham *et al.* 2016) (Fig. 2F).

In studies using looped solution DNA probes (Reid *et al.* 2015; Zhao *et al.* 2019), the immobilized DNA was tagged with the acceptor (Cy5), and the free end of the looped DNA was tagged with a donor (Cy3). A 532-nm laser was used to excite the imaging slide. When DNA synapsis occurred, both donor and acceptor signals appeared (Figs. 2B and 2D). The appearance of a FRET level around 0.8 as that of the pre-ligated control indicated the formation of close synaptic (CS) complexes, where the two duplexes were in end-to-end configuration (Figs. 2D and 2E). Lower FRET levels suggested the formation of flexible synaptic (FS) complexes with side-by-side duplex alignment (Fig. 2C), while transitions from low to high FRET indicated FS to CS transitions (Fig. 2D). In studies using two-acceptor labeled DNA probes (Graham *et al.* 2016) (Fig. 2F), two lasers—532 nm and 641 nm were applied to illuminate the slide (Figs. 2F and 2G). Colocalization of donor and acceptor signals indicated synapsis of two duplexes. No or low FRET signal suggested the formation of a synaptic complex with two duplexes in a loose state, while high FRET indicated that the two ends of the duplexes were in close contact within the synaptic complex (Figs. 2F and 2G).

The intramolecular DNA probe setup involves as long as a 2-kb DNA fragment with a donor and acceptor labeled at each end and a biotin tag in the center for immobilization (Graham *et al.* 2016) (Fig. 2H). The appearance of a high FRET signal indicates the formation of a synaptic complex with two ends in a close contact state (Figs. 2H and 2I). Although this intramolecular type can yield higher synapsis efficiency compared to intermolecular studies, intramolecular synapsis is less reflective of the actual repair mechanisms in cells, where chromosomal breaks typically arise from two different chromosomes rather than the same one. And even for breaks in the same chromosome, the large chromosome size (in Mb) makes the two ends less resemble the intramolecular type (in kb) (Li and Tyler 2016).

### The mechanical method for studying NHEJ synapsis

**Figure 3:**
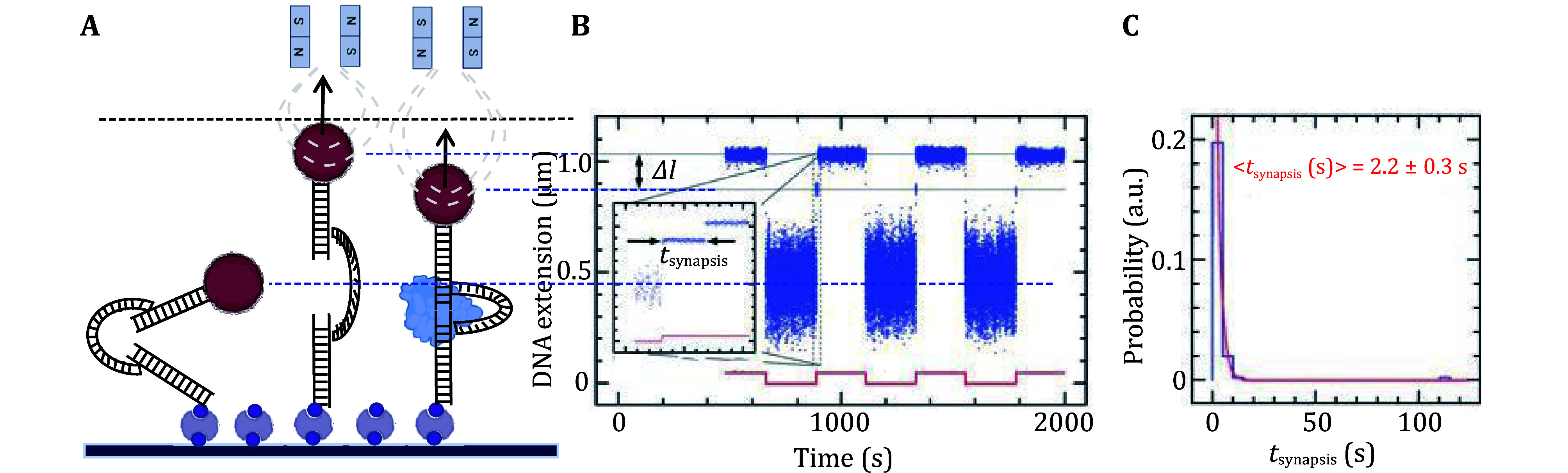
Single-molecule magnetic tweezers for studying NHEJ synapsis. This figure illustrates how single-molecule magnetic tweezers is utilized to investigate the synapsis process in non-homologous end joining (NHEJ). **A** The magnetic tweezers assay uses a novel DNA construct. One long DNA strand is attached to the surface of a flow cell, while another long DNA is connected to a magnetic bead. A short linker DNA is covalently attached to the free ends of both long DNA strands. By cycling between low and high forces, real-time changes in DNA extension can be observed. **B**,**C** When synapsis occurs, a transient and relatively stable period of DNA extension is observed under high force. The lifetime of the formed synaptic complex can be deduced from the transient plateau formed during DNA extension under high force. Adapted from Wang *et al*. 2018

The primary mechanical method for studying NHEJ synapsis is the magnetic tweezers technique (De Bragança *et al.* 2023a; Wang *et al.* 2018). Magnetic tweezers use an external magnetic field generated by magnets to manipulate paramagnetic microspheres. In this setup, DNA molecules are attached to these microspheres, with one end connected to a magnetic bead and the other to the surface of a flow cell (Fig. 3A). By adjusting the magnetic field vertically, the DNA can be stretched, allowing real-time monitoring the formation and disruption of synaptic complexes, thus enabling the determination of the thermodynamics and lifetimes of the synaptic complexes (De Bragança *et al.* 2023a; Wang *et al.* 2018) (Fig. 3).

The DNA probes used in magnetic tweezer assays to study NHEJ synapsis are ingeniously designed (De Bragança *et al.* 2023a; Wang *et al.* 2018). Two linear dsDNA fragments (~1.5 kb) are connected by covalently linking them to a third dsDNA fragment (~700 bp). The ends of this short dsDNA fragment are positioned about 60 bp from the ends of each longer dsDNA, providing enough space for NHEJ protein assembly (Fig. 3A). Synapsis between the 60 bp ends can occur under low-force conditions, and is disrupted when higher force is applied (Figs. 3A and 3B). Synapsis formation results in a transient plateau in the DNA extension signal, which is observed as the force is increased (Fig. 3B). The lifetime of the synaptic complex can be deduced from the duration of this plateau (Figs. 3B and 3C). By cycling between the low and high forces, multiple synapsis events can be recorded from a single DNA construct (Fig. 3B), allowing the lifetime of a specific synaptic complex to be obtained with minimal DNA constructs (Fig. 3C).

This magnetic tweezers assay provides insights into the essential NHEJ proteins required to form observable synaptic complexes, as well as the effects of each protein on the duration of these complexes. However, the spatial resolution of magnetic tweezers is limited, preventing detailed information about the structural arrangements of the DNA ends, as can be obtained with smFRET. Additionally, the dsDNA linker within the construct can bind NHEJ proteins, such as XLF and XRCC4, potentially interfering with the assembly of these proteins on the shorter dsDNA ends and thus affecting synapsis. Neutral polymers like polyethylene glycol (PEG) (Bel 2023), which can serve as an alternative linker, may offer better results than dsDNA linkers for studying NHEJ synapsis with magnetic tweezers.

## RECENT PROGRESS ON THE MOLECULAR BASIS OF NHEJ SYNAPSIS UNVEILED BY SINGLE-MOLECULE ASSAYS

The high spatiotemporal resolution of single-molecule techniques allows the identification of several synaptic intermediates (Graham *et al.* 2016; Reid *et al.* 2015; Stinson *et al.* 2020; Zhao *et al.* 2019, 2020b). These studies have revealed the arrangements of the two dsDNA within synaptic complexes, the specific NHEJ proteins required for synapsis, and the transitions between synaptic states. smFRET assays have provided structural insights by classifying synaptic complexes based on the spatial arrangements of the DNA ends (Graham *et al.* 2016; Reid *et al.* 2015; Stinson *et al.* 2020; Zhao *et al.* 2019, 2020b). In contrast, magnetic tweezers assays categorize synaptic complexes by their energetics, focusing on their stability and lifetime (De Bragança *et al.* 2023a; Wang *et al.* 2018).

### Synapsis mediated by core NHEJ proteins

**Figure 4:**
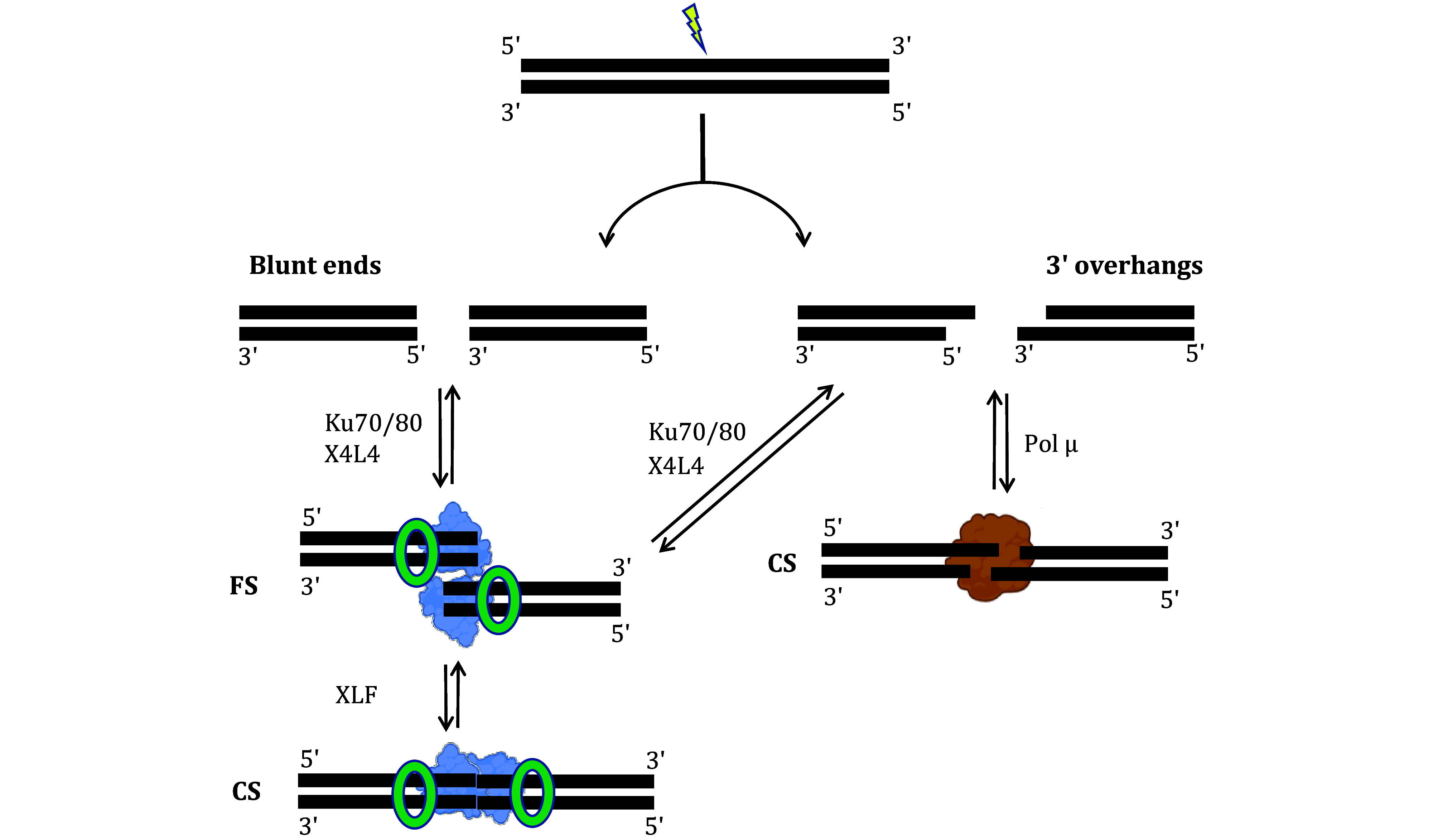
Mechanisms of NHEJ synapsis. This figure explains the mechanisms underlying NHEJ synapsis. Double-strand break (DSB) ends can vary, including blunt ends and 3' overhangs. For blunt ends, Ku70/80 and X4L4 mediate a flexible synapsis (FS), where two dsDNAs are arranged laterally. XLF can then facilitate the transition of the FS complex into a close synaptic (CS) complex, characterized by an in-line end-to-end configuration of the two dsDNAs. For 3’ overhangs, if the DNA overhangs contain at least one base pair of 3' microhomology, pol μ alone can mediate the synapsis

The core NHEJ proteins—Ku70/80, XRCC4-Ligase4 (X4L4), and XLF—were initially reported to mediate synapsis via a filament structure (Hammel *et al.* 2010). However, the filament model did not fully explain the interactions between the DNA ends. The Lieber and Rothenberg labs, using smFRET with purified NHEJ proteins and blunt-ended DNA (Figs. 2A–2E), provided a clearer picture (Zhao *et al.* 2019). Their work showed that Ku70/80 and X4L4 are sufficient to mediate a flexible synaptic (FS) state, where the two DNA molecules are aligned side by side (Zhao *et al.*, 2019) (Figs. 2A–2E and Fig. 4). XLF facilitates the transition from FS to a close synaptic (CS) state (Zhao *et al.* 2019), in which the DNA ends are positioned end-to-end, allowing for ready ligation (Figs. 2A–2E and Fig. 4). In contrast, the FS state might permit the ends to have sufficient space for end processing, but without letting the ends diffuse apart.

Interestingly, DNA-PKcs was found to be non-essential for NHEJ synapsis in purified systems, as shown by smFRET studies (Conlin *et al.* 2017; Reid *et al.* 2015, 2017; Zhao *et al.* 2019, 2020b). While DNA-PKcs may play a role in initial synapsis and transitions between states in more complex systems (*e*.*g*., Xenopus egg extracts containing histones and hundreds of other unidentified proteins) (Graham *et al.* 2016) (Figs. 2F–2I), it does not significantly impact the FS or CS complexes in purified settings (Zhao *et al.* 2019). Furthermore, the CS complex can form directly using purified Ku70/80, X4L4, and XLF proteins but without DNA-PKcs in a structural study (Chen *et al.* 2021), highlighting its non-essential roles in the formation of and transition to the CS complex. These findings align with observations from V(D)J recombination (Gao *et al.* 1998; Jiang *et al.* 2015; Kulesza and Lieber 1998), where DNA-PKcs is not required for signal joint formation. It is very possible that in crude extract systems (Graham *et al.* 2016; Stinson *et al.* 2020), DNA-PKcs is needed in the initial phases of NHEJ to displace non-NHEJ proteins from the DNA ends to enable synapsis. Of course, DNA-PKcs is essential in the processing phase of NHEJ to activate Artemis for nuclease action (Lu *et al.* 2008).

### Synapsis mediated by DNA polymerase mu (pol μ)

Pol μ plays a critical role in end processing during NHEJ repair, with its template-dependent synthesis suggesting that it may also mediate synapsis (Andrade *et al.* 2009; Davis *et al.* 2008). A study by the Lieber laboratory, using smFRET, directly detects NHEJ synapsis by pol μ (Zhao *et al.* 2020b). Their findings revealed that pol μ alone could mediate synapsis (Zhao *et al.* 2020b) (Fig. 4) but only when the DNA overhangs have at least one base pair of 3’ overhang microhomology. In this synaptic complex, the overhangs are aligned in a physiological configuration that allows pol μ to add nucleotides to the upstream primer, enabling subsequent ligation by X4L4 (Zhao *et al.* 2020b) (Fig. 4). This alternative synapsis mechanism differs from that driven by the core NHEJ proteins (Ku70/80, X4L4, and XLF), providing further evidence of the flexibility and complexity of the NHEJ synapsis.

### Diverse mechanisms of NHEJ synapsis

Besides the mechanisms discovered by smFRET assays, magnetic tweezers have also identified multiple intermediates of synaptic complexes, categorized by their stability (De Bragança *et al.* 2023a; Wang *et al.* 2018). The Strick laboratory developed a magnetic tweezers assay (Fig. 3) that proposes a stepwise assembly model of NHEJ synapsis (Wang *et al.* 2018). In this model, Ku70/80 and DNA-PKcs form an initial synaptic complex that lasts about 100 milliseconds. This intermediate is further stabilized by X4L4, XLF, and/or PAXX, leading to the formation of more stable synaptic complexes, which can persist for seconds to minutes (Wang *et al.* 2018). Interestingly, the roles of DNA-PKcs and PAXX in stabilizing the synaptic complexes differ from observations in studies using smFRET method (Reid *et al.* 2015; Zhao *et al.* 2019), indicating potential discrepancies between techniques. These differences may arise from variations in experimental systems, such as the influence of linker dsDNA within the constructs on NHEJ protein binding (Fig. 3A).

Another study, using a similar DNA construct, identified new players in NHEJ synapsis, including APLF and the long non-coding RNA (IncRNA) NIHCOLE (De Bragança *et al.* 2023a), further demonstrating the diversity and flexibility of the synapsis step in NHEJ (Chang *et al.* 2017; Pannunzio *et al.* 2018).

### The coupling between synapsis and end-processing

The interplay between end synapsis and processing in NHEJ is an area of particular focus in recent studies, revealing that these steps are more interconnected than previously thought (Stinson *et al.* 2020; Zhao *et al.* 2019, 2020b). The precise timing of end processing relative to synapsis is a key question that single-molecule assays have started to address.

Traditional bulk studies have suggested that end processing is iterative, with DNA ends cycling between processing and ligation steps (Chang *et al.* 2017; Chang *et al.* 2016; Ma *et al*. 2005; Ma *et al.* 2004; Pannunzio *et al.* 2018; Simsek and Jasin 2010). However, these assays have not determined whether processing occurs before, during, or after synapsis. One recent smFRET study using Xenopus egg extract has shown that the processing of DNA ends by pol λ-mediated fill-in and the TDP1-mediated damage-removal occurs only when the two ends are in a close synaptic state (Stinson *et al.* 2020). This finding suggests that the final stages of synapsis must be completed before certain end-processing steps can occur. However, this smFRET study could not establish whether the critical end-processing proteins, Artemis and pol μ, act before or during synapsis (Stinson *et al.* 2020). A cellular study, meanwhile, suggests that end processing occurs in a relaxed and loosened synaptic state rather than a close state (Buehl *et al.* 2023), which contradicts the smFRET study (Stinson *et al.* 2020). The discrepancies between these findings highlight the need for further single-molecule investigations to clarify how to end processing and synapsis are coordinated in real time.

## SUMMARY AND PERSPECTIVES

Single-molecule techniques, with their unparalleled spatial and temporal resolution, have significantly advanced our understanding of NHEJ synapsis. These methods have uncovered multiple synaptic intermediates and highlighted the diverse mechanisms by which NHEJ proteins coordinate the repair process. However, some discrepancies remain between the conclusions drawn from different single-molecule techniques, such as smFRET and magnetic tweezers, which may be due to differences in the experimental systems used.

One major challenge in the field is to reconcile the findings from studies using purified systems with those using cellular extracts. While purified systems allow for the precise dissection of the roles of individual NHEJ proteins, cellular studies may reveal additional complexities introduced by non-NHEJ components. Further research using a combination of these approaches is necessary to fully understand the NHEJ process.

Additionally, future studies should focus on developing new single-molecule methods, such as single-molecule multi-color fluorescence-colocalization/FRET assays and single-molecule fluorescence coupled forceps assays (Ghimire and Guo 2021; Ma *et al.* 2021; Zhang *et al.* 2021; Zuo *et al*. 2022), to investigate the coupling between synapsis and end processing, with particular focus on the roles of essential end-processing enzymes such as Artemis and pol μ. Understanding the timing and sequence of these events is critical for a complete picture of NHEJ repair.

In conclusion, while much progress has been made in elucidating the molecular mechanisms of NHEJ synapsis, further work is needed to bridge the gaps between different experimental approaches and to fully explore the coupling between synapsis and the other steps in NHEJ. With continued advancements in single-molecule techniques, we are poised to gain even deeper insights into the complexities of DNA repair processes.

## Conflict of interest

Yuhao Jiang, Chao Zhao, Chenyang Zhang, Weilin Li, Di Liu and Bailin Zhao declare that they have no conflict of interest.
